# Understory Dwarf Bamboo Affects Microbial Community Structures and Soil Properties in a *Betula ermanii* Forest in Northern Japan

**DOI:** 10.1264/jsme2.ME16154

**Published:** 2017-04-28

**Authors:** Bihe Kong, Lei Chen, Yasuhiro Kasahara, Akihiro Sumida, Kiyomi Ono, Jan Wild, Arata Nagatake, Ryusuke Hatano, Toshihiko Hara

**Affiliations:** 1 Institute of Low Temperature Science, Hokkaido UniversityKita-19, Nishi-8, Kita-ku, Sapporo 060–0819Japan; 2 Soil Science Laboratory, Graduate School of Agriculture, Hokkaido UniversityKita-9, Nishi-9, Kita-ku, Sapporo, 060–8589Japan

**Keywords:** *Sasa kurilensis*, fungi, bacteria, boreal forest, high-throughput sequencing

## Abstract

In order to understand the relationships between understory bamboo and soil properties, we compared microbial community structures in the soil of a *Betula ermanii* boreal forest with *Sasa kurilensis* present and removed using high-throughput DNA sequencing. The presence of understory *S. kurilensis* strongly affected soil properties, including total carbon, total nitrogen, nitrate, and the C:N ratio as well as relative soil moisture. Marked differences were also noted in fungal and bacterial communities between plots. The relative abundance of the fungal phylum *Ascomycota* was 13.9% in the *Sasa*-intact plot and only 0.54% in the *Sasa*-removed plot. Among the *Ascomycota* fungi identified, the most prevalent were members of the family *Pezizaceae*. We found that the abundance of *Pezizaceae*, known to act as mycorrhizal fungi, was related to the amount of total carbon in the *Sasa*-intact plot. The relative abundance of *Proteobacteria* was significantly higher, whereas those of *Planctomycetes* and *Actinobacteria* were lower in the *Sasa*-intact plot than in the *Sasa*-removed plot. Furthermore, the results obtained suggest that some species of the phylum *Planctomycetes* are more likely to occur in the presence of *S. kurilensis*. Collectively, these results indicate that the presence of *S. kurilensis* affects microbial communities and soil properties in a *B. ermanii* boreal forest.

Understory dwarf bamboos (genus *Sasa*; clonal evergreen plants) are widely distributed in the boreal forests of northern Japan (19, 41, 44, 46). In heavy snowfall areas, dwarf bamboos may quickly cover forests, growing to 0.3–3 m in height with vigorously extending rhizomes (33, 40). Dense dwarf bamboo cover impacts the regeneration of tree species and affects tree seedling growth and establishment (22, 30). Since the mass of bamboo’s intertwining roots forms rhizomes underground, understory dwarf bamboo is considered to compete with overstory plants for soil nutrients and water (28, 32, 37). Previous studies demonstrated that *Sasa senanensis* (Franch. & Sav.) Rehder plays an important role in mitigating the loss of nitrogen and carbon from soil (13). The removal of *Sasa* spp. from a mixed boreal forest was previously shown to affect the dynamics of dissolved inorganic nitrogen in soil (35) and promote nitrogen leaching (12). Moreover, the removal of *Sasa kurilensis* (Rupr.) Makino & Shibata has been suggested to significantly increase the availability of soil water and enhance the growth of *Betula ermanii* Cham. trees (41). These findings indicate that understory *Sasa* sp. is closely linked to the properties of its surrounding soil.

In nutrient-poor ecosystems, soil microbes are crucial regulators of plant productivity (49). Variations in the structures of soil microbial communities are considered to be a major factor affecting litter decomposition rates (39). A methanotrophic bacterial community called the upland soil cluster alpha was found to have a close relationship with *Sasa* spp. in boreal forests (47). Moreover, *Sasa* spp. may be infected by witches’ broom disease through their fungal communities (31, 42). Species-species information on endophytic fungi in *Sasa* spp. has been expanded (29). However, the complexity of microbial communities in forest habitats makes it difficult to elucidate the species composition of soil microorganism communities under natural conditions. To date, the entire microbial community in the rhizosphere soil of *Sasa* spp. as a typical understory plant in boreal forests has not yet been investigated.

Rapid advances in molecular ecological approaches have made it possible to analyze whole microbial communities in a relatively short time. In particular, Illumina MiSeq has been widely used in many studies and has the advantage of allowing a higher sequencing depth than the 454 pyrosequencing technique (4, 38, 48). In the present study, we used Illumina MiSeq to analyze the microbial community structures of soils with or without understory *S. kurilensis* in a *Betula ermanii* stand and attempted to clarify the relationships between community structures and soil physicochemical properties. The main objectives of this study were to answer the following questions: (i) what are the structures of microbial communities in the presence and absence of *S. kurilensis*? and (ii) how are these structures related to soil physicochemical properties?

## Materials and Methods

### Study site and sample collection

This study was conducted in the Uryu Experiment Forest of Hokkaido University (44°23′N, 142°19′E) in northern Japan. The tree layer of the forest was exclusively dominated by the birch species, *B. ermanii*, and the forest floor was densely covered with *S. kurilensis*. Two plots of 20×30 m were established in this forest in 1998 (19, 41). *S. kurilensis* has been removed continuously since 1998 in one plot (*Sasa*-removed plot; SR) and left intact in the other plot (*Sasa*-intact plot; SI). The distance between the two plots was approximately 50 m.

Soil temperature and moisture were monitored using TOMST thermometers (TOMST Ltd., Prague, Czech Republic). Three temperature readings (air temperature at 10 cm above the soil surface, soil surface temperature, and 15-cm-deep soil temperature) and soil moisture were recorded every 15 min. A TOMST thermometer measuring system was set at the center of each circle (16, 18, 24).

**Fig. 1:**
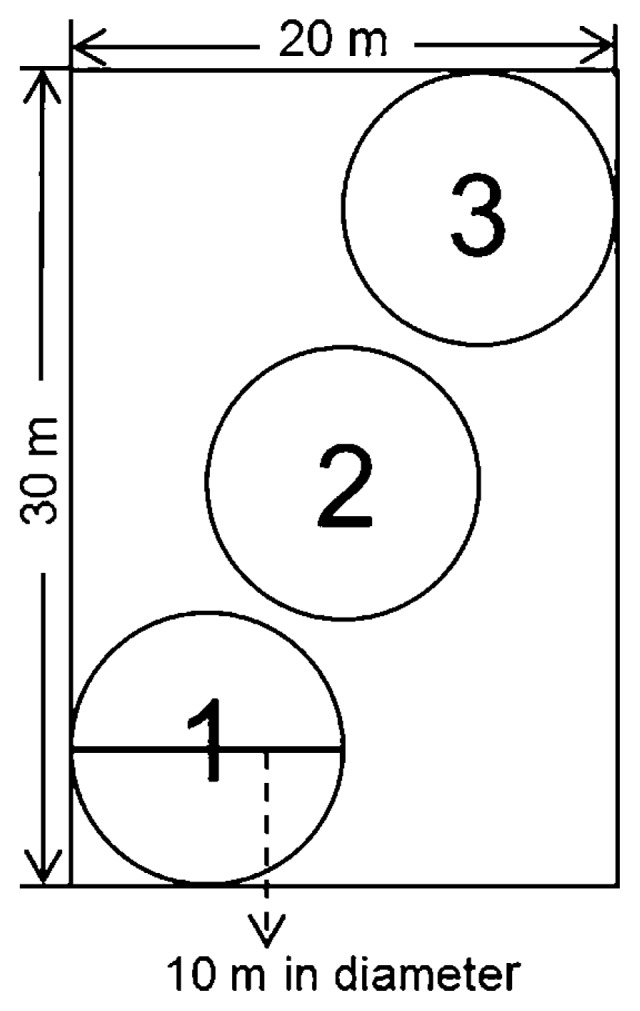
Locations of three sampling circles used in both study plots (*Sasa*-intact and *Sasa*-removed).

Soil samples were collected 5 times during the growing season: on 23 July 2014, 1 October 2014, 3 June 2015, 4 August 2015, and 1 October 2015, by avoiding the snowy season. Three clods of soil (1,000 cm^3^) were randomly collected at a depth of 0–10 cm from each of the three fixed circles (10 m in diameter) on a diagonal line in the two plots (Fig. 1). In the SI plot, soil samples including *S. kurilensis* rhizomes were shaken vigorously to separate roots and soil not tightly adhering to roots. Soil samples of three replicates from each circle were mixed as one sample, packed in sterile plastic bags (Ziploc; SC Johnson Co., Racine, WI, USA), and transported to the laboratory in an ice-cooled box. After being sieved through a 2-mm screen, each soil sample was divided into three parts and stored in a −80°C refrigerator, a 4°C refrigerator, or a dry, room temperature location, respectively.

### Soil property analysis

Thirty soil samples (5 sampling dates×2 plots×3 circles) from the two plots were collected for soil property analysis. Soil pH was measured using a glass electrode after shaking with deionized water (1:2.5 soil: water ratio) for 1 h. Total nitrogen (TN) and total carbon (TC) contents in air-dried soil samples were measured using an automatic analyzer (C-N Corder; Yanaco, Kyoto, Japan). In order to measure inorganic nitrogen, fresh soil samples were mixed with deionized water (1:5 soil:water) for nitrate nitrogen (NO_3_-N) and nitrite nitrogen (NO_2_-N), and 2M KCl (1:10 soil:KCl) for ammonium nitrogen (NH_4_-N). The concentrations of NO_3_-N and NO_2_-N were measured with ion chromatography (Dionex Model ICS-1100; Thermo Fisher Scientific, Waltham, MA, USA) and ammonium using the indo-phenol blue method (20).

### DNA extraction and library preparation

A PowerSoil™ DNA Isolation Kit (MO BIO Laboratories, Solana Beach, CA, USA) was used to extract DNA from 0.25 g of fresh soil samples according to the manufacturer’s instructions. The genomic DNA standard of each sample was greater than 200 ng and the concentration of DNA was greater than 6 ng μL^−1^ without degradation, after testing with a Beckman DU800 spectrophotometer (Beckman Coulter, Inc., Fullerton, CA, USA). DNA isolated from each sample was amplified using forward Bakt_341F (5′-CCTACGGGNGGCWGCAG-3′) and reverse Bakt_805R (5′-GACTACHVGGGTATCTAATCC-3′) primers for the V3-V4 region of the bacterial 16S rRNA gene (15). Forward ITS3 (5′-GCATCGATGAAGAACGCAGC-3′) and reverse ITS4 (5′-TCCTCCGCTTATTGATATGC-3′) primers were also used for fungal ITS genes (1).

Fifteen sequencing libraries were prepared per plot with the random fragmentation of DNA samples, followed by 5′ and 3′ adapter ligation. Adapter-ligated fragments were then used for PCR, and gel amplicons were purified according to the Illumina library preparation protocol. DNA sequencing was performed by Macrogen Inc. (Seoul, Korea) using the Illumina Miseq platform (San Diego, CA, USA) according to the manufacturer’s instructions.

### Sequence process and statistical analyses

Raw data formatted as FASTQ were analyzed with FLASH version 1.2.11 to merge paired-end 300-bp reads from next-generation sequencing experiments (26). The CD-HIT-OTU program (http://weizhong-lab.ucsd.edu/cd-hit-otu/) was used to identify operational taxonomic units (OTUs) (25). Three-step clustering was performed with (1) raw read filtering and trimming; (2) error-free read selection; and (3) OTU clustering at distant cut-offs (0.03). The taxonomy assignment of bacterial and fungal sequences was based on the full alignments of 16S rRNA and ITS genes, respectively. Cluster sequences were uploaded to the Silva SSU database for 16S rRNA genes and the UNITE database for ITS genes. Quantitative insights into microbial ecology (QIIME) software version 1.9.1 were used to perform alpha diversity analyses (Chao1, Shannon Index, Simpson Index) on bacterial and fungal communities (3). Differences in the number of OTUs and alpha diversity estimates were tested using a one-way ANOVA, followed by Tukey’s honest significant difference test (HSD).

Significant differences in soil physicochemical properties between the SI and SR plots on the five sampling dates were tested using generalized linear mixed models, followed by Tukey’s HSD test. Generalized linear mixed models were also used to examine the relationships between the number of OTUs of microbial communities and soil physicochemical properties in the SI and SR plots. Differences between the relative abundance of microbial communities were tested with the Kruskal–Wallis method (21). All statistical calculations were performed in R version 3.2 (36). Values *p*<0.05 were considered to be significant. A redundancy analysis (RDA) is a constrained ordination technique that was used to test whether the occurrence of microbial communities matches specific environmental source profiles (8, 27), which was performed with abundance and environmental data using Canoco version 4.5 (43).

## Results

### Soil characteristics

**Fig. 2:**
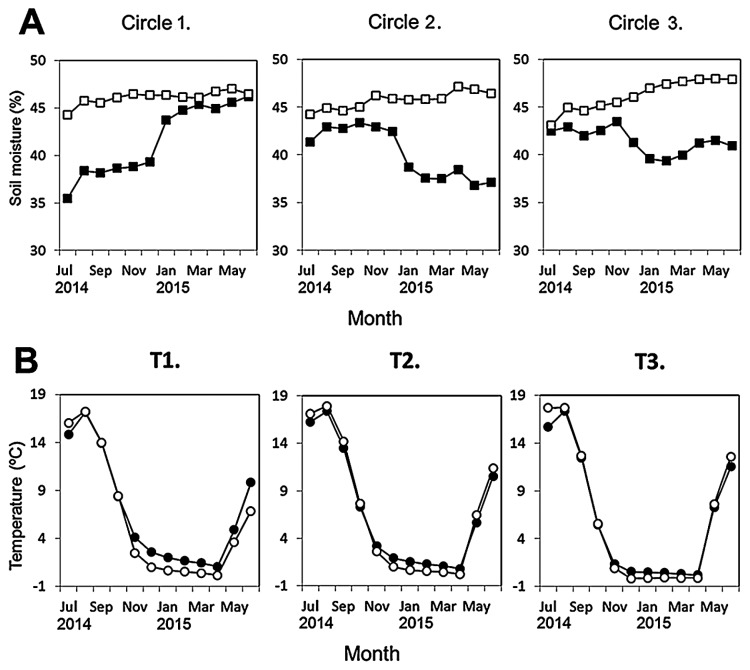
Monthly mean (A) soil moisture (squares) and (B) temperature (circles) of three sampling circles in *Sasa*-intact (filled) and *Sasa*-removed (open) plots between July 2014 and June 2015. T1: air temperature; T2: soil surface temperature; T3: 15-cm-deep soil temperature.

**Fig. 3:**
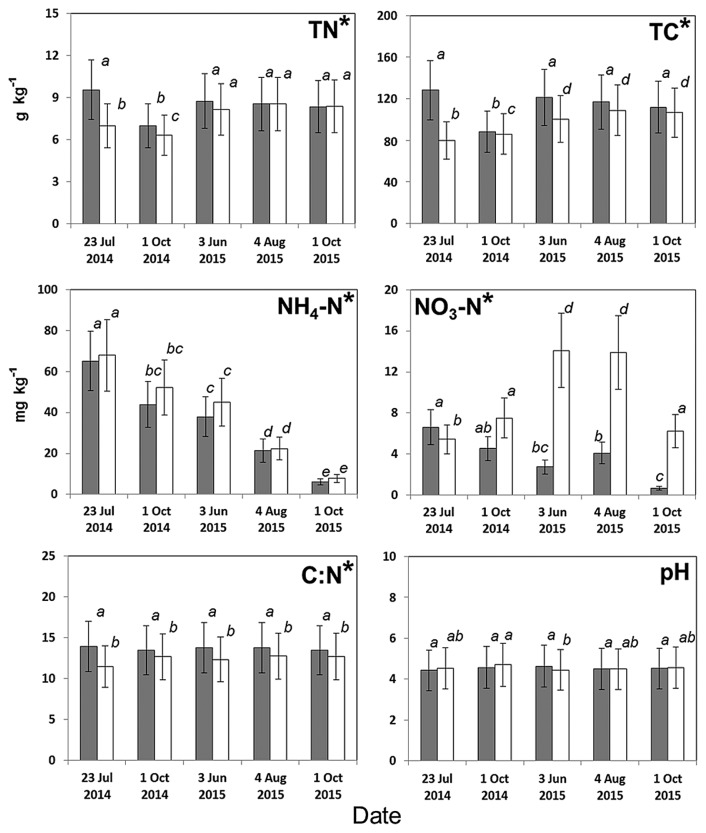
Differences in soil physicochemical properties between *Sasa*-intact (gray) and *Sasa*-removed (white) plots according to a generalized linear mixed model. The superscript asterisk indicates significance at *p*<0.05. The response variable was the concentration of soil properties, the fixed effect was the two study plots, and the random effect was the five sampling dates. Mean values with different letters denote significant differences among the pooled data (*i.e.*, data for five sampling dates for both plots) (*p*<0.05; Tukey’s HSD, *n*=9). TN: total nitrogen; TC: total carbon; NH_4_-N: ammonium; NO_3_-N: nitrate. Vertical lines indicate standard errors.

Significant differences were observed in most of the physicochemical soil properties tested between the SI and SR plots. Soil moisture was markedly lower in the SI plot than in the SR plot (Fig. 2A). Although no significant difference was observed in soil temperatures (Fig. 2B) or pH, all other values were significantly different between the two plots (Fig. 3). Total carbon and the C:N ratio were higher in the SI plot than in the SR plot, while ammonium was lower, regardless of the sampling dates. The nitrate N concentration was significantly different between the SI and SR plots for all sampling dates, except 1 Oct 2014. Nitrite N was not detected (<1 mg NO_2_-N kg^−1^ soil) during the sampling period (data not shown).

### Abundance of 16S and ITS sequences

**Fig. 4:**
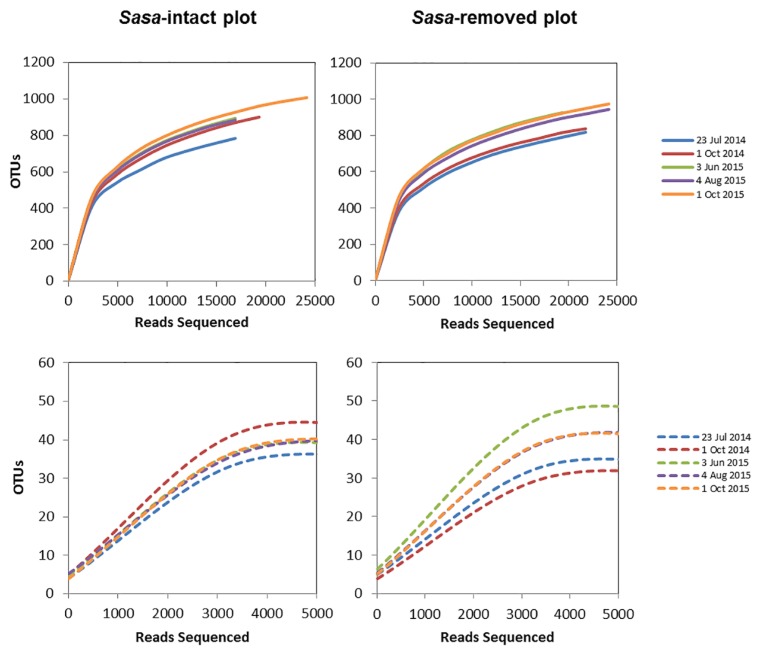
Rarefaction curves for microbial OTUs in the three circles on five sampling dates, with clustering at a 97% rRNA sequence similarity. Solid and dashed lines represent the OTUs of the bacterial and fungal communities, respectively.

After multiple levels of quality control to filter the raw reads, we obtained an average of 26,665 sequences for the 16S rRNA genes and an average of 31,080 sequences for the ITS genes from all samples. All rarefaction curves were generated via CD-HIT-OTU using a 97% identity cut-off; coverage was more than 99% for all the samples. These results indicated that the volume of sequenced reads was reasonable and sufficient to evaluate the total number of OTUs (Fig. 4). Most bacterial samples were saturated at approximately 720–1,125 OTUs, whereas fungal samples were saturated at approximately 27–55 OTUs. Only the Shannon diversity index for alpha diversity estimates of microbial communities for 23 July 2014 was significantly different between the SI and SR plots (Table 1).

**Fig. 5:**
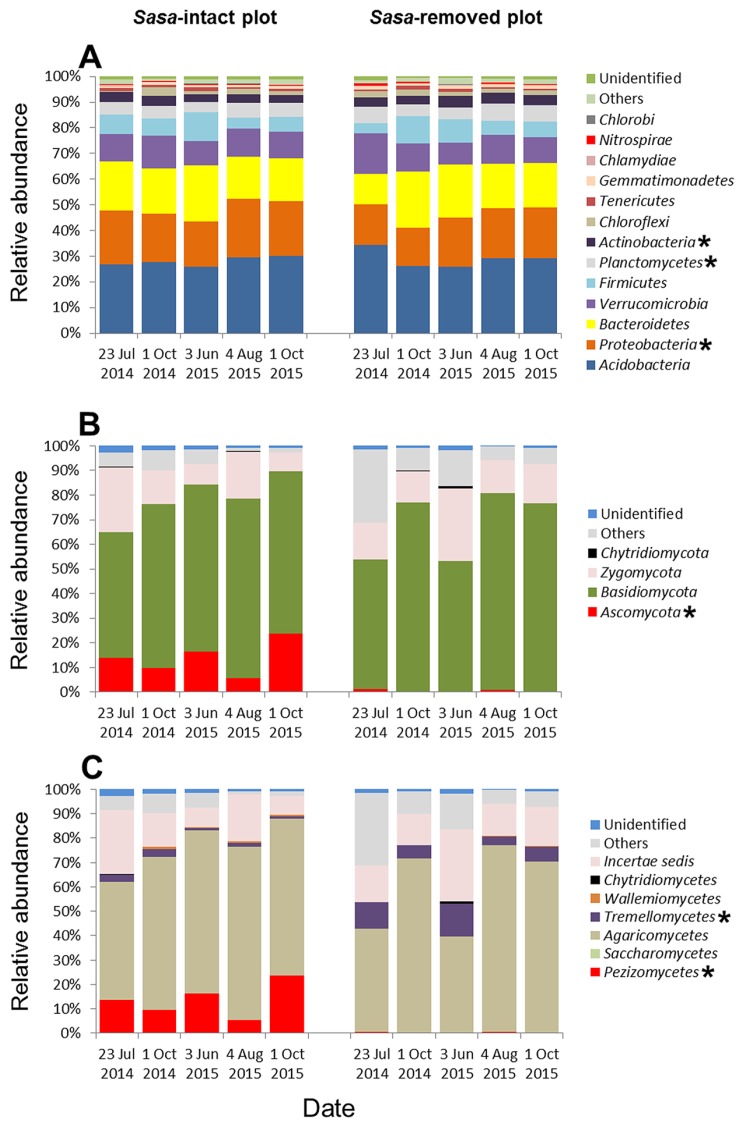
Differences in the relative abundance of soil microbial communities in *Sasa*-intact and *Sasa*-removed plots for five sampling dates according to a Kruskal–Wallis test. The asterisk indicates significance (*p*<0.05; *n*=15). (A) Bacterial phyla; (B) Fungal phyla; (C) Fungal classes.

Bacterial OTUs were assigned to 13 different phyla. Seven different phyla (*Acidobacteria*, *Proteobacteria*, *Bacteroidetes*, *Verrucomicrobia*, *Firmicutes*, *Planctomycetes*, and *Actinobacteria*) together comprised more than 90% of the relative abundance in every library (Fig. 5A). Since each of the six other phyla comprised <1% in both the SI and SR plots, they were excluded from further analyses. *Proteobacteria* (20.3% in the SI plot and 17.9% in the SR plot), *Planctomycetes* (4.99% and 5.71%), and *Actinobacteria* (3.43% and 4.01%) were tested using the non-parametric Kruskal–Wallis method, which revealed significant differences between the two plots. The other phyla were not significantly different between the plots: *Acidobacteria* (28.0% in the SI plot and 29.0% in the SR plot), *Bacteroidetes* (18.4% and 17.7%), *Verrucomicrobia* (10.8% and 11.2%), and *Firmicutes* (7.11% and 7.09%). The seven dominant phyla occurred in soil irrespective of the presence of *S. kurilensis*. Overall, the number of OTUs of fungal communities was fewer than those of bacterial communities.

Fungal sequences belonged to four different phyla: *Ascomycota*, *Basidiomycota*, *Zygomycota*, and *Chytridiomycota* (Fig. 5B). *Basidiomycota* had an average abundance of 64.9% in the SI plot and 67.8% in the SR plot over all the sampling dates. *Zygomycota* comprised 15.0% and 17.2% in the SI plot and SR plot, respectively. *Chytridiomycota* was excluded from further analyses because abundance was <1% in both plots. Notably, *Ascomycota* had a very different abundance between the two plots, making up 13.9% of the SI plot, but only 0.54% of the SR plot. The relative abundance of the fungal class *Pezizomycetes* (phylum *Ascomycota*) in the SI plot was greater than that in the SR plot (Fig. 5C), while *Tremellomycetes* (phylum *Basidiomycota*) was less abundant in the SI plot than in the SR plot for all sampling dates. Most of the abundances in the phyla *Ascomycota* and *Basidiomycota* were attributed to the family *Pezizaceae* and genus *Cryptococcus*, respectively (Table 2).

### Relationships between microbial communities and soil properties

A generalized linear mixed model was used to test whether environmental factors were related to the relative abundances of microbial species. In Table 3, *Firmicutes* had a close relationship with NH_4_-N concentrations and *Planctomycetes* was strongly related to TC and TN contents in the SI plot. In the SR plot, the bacterial phylum *Firmicutes* was related to soil pH, *Proteobacteria* was related to TN contents, and the fugal phylum *Basidiomycota* was related to NH_4_-N concentrations.

**Fig. 6:**
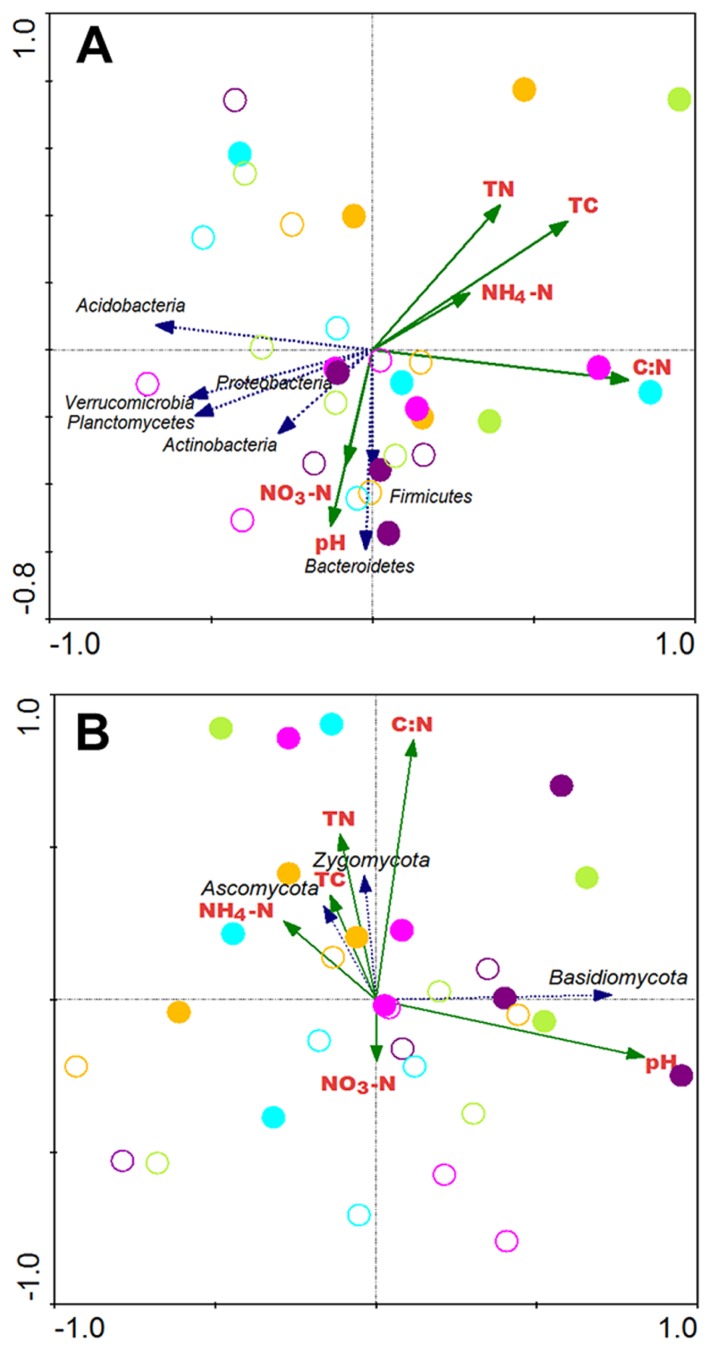
Redundancy analysis of main (A) bacterial and (B) fungal communities in *Sasa*-intact (solid circles) and *Sasa*-removed (open circles) plots based on Canoco 4.5. The purple, light green, orange, aqua, and pink colors represent the sampling dates of 23 July 2014, 1 October 2014, 3 June 2015, 4 August 2015, and 1 October 2015, respectively. Blue and dark green arrows indicate the abundance of microbes and values of soil properties, respectively. TN: total nitrogen; TC: total carbon; NH_4_-N: ammonium; NO_3_-N: nitrate.

RDA has generally been used in microbial ecology to examine whether variations in microbial taxa changes correspond to environmental characteristics. As shown in Fig. 6A, phyla *Bacteroidetes* and *Firmicutes* were related to NO_3_-N concentrations or pH, and five other bacterial phyla were each strongly and negatively related to the TN content, TC content, NH_4_-N concentration, or C:N value in the SI plot. Although the species richness of fungi was low, the RDA analysis showed that the fungal phylum *Basidiomycota* was closely related to pH. Phyla *Ascomycota* and *Zygomycota* were both positively related to the TN content, TC content, NH_4_-N concentration, and C:N value in the SI plot, but negatively related to the NO_3_-N concentration in the SR plot (Fig. 6B).

## Discussion

### Microbial community in the presence of *S. kurilensis*

We found that the abundance of mycorrhizal fungi of the phylum *Ascomycota* was markedly higher in the SI plot (13.9%) than in the SR plot (0.54%) (Fig. 5B). Members of the family *Pezizaceae* were predominant among the *Ascomycota* identified in this study (Table 2); some *Pezizaceae* species are known to form truffles, while others act as mycorrhizal fungi (14, 23). These findings suggest that *S. kurilensis* serves as a host to a large reservoir of bamboo-associated mycorrhizal fungi. In the present study, the relative abundances of the main bacterial phyla identified (*Proteobacteria*, *Planctomycetes*, and *Actinobacteria*) were significantly different between the SI and SR plots (Fig. 5A), and the phyla *Planctomycetes* and *Actinobacteria* were slightly more abundant in the *Sasa*-removed plot than in the *Sasa*-intact plot (Fig. 6A). In soil, *Proteobacteria*, *Planctomycetes*, and *Actinobacteria* were found to be the main bacterial phyla co-occurring with the fungal class *Pezizomycetes* in the United States at almost the same latitude as our study site (50). Moreover, in the same study plots, Tsutsumi *et al.* found that the presence of *S. kurilensis* was closely related to the composition of methanotrophic bacterial communities (47). This result is consistent with our finding that the rhizome soil of *S. kurilensis* had a significant effect on the composition of bacterial communities (in addition to fungal ones). Therefore, we hypothesized that the presence of *S. kurilensis* affected the fungal family *Pezizaceae* (phylum *Ascomycota*) and bacterial phyla *Proteobacteria*, *Planctomycetes*, and *Actinobacteria*.

### Microbial community structures may mediate soil properties

Due to their rich organic matter and low microbial decomposition rate, boreal forests act as a terrestrial net carbon sink in which a large amount of carbon is stored in tree roots and root-associated microorganisms (5, 34). Previous studies reported that the C:N ratio is a good indicator of microbial community structures in boreal forests (17). In the present study, we showed that the values of TC, TN, and C:N differed between the SI and SR plots (Fig. 3). Tripathi *et al.* (44, 45) reported differences in soil properties and nitrogen availabilities among bamboo stands including the SI and SR plots used in our study. These findings suggest that the composition of microbial communities, *S. kurilensis*, and soil properties in *Betula* forests are closely related, possibly because of their interactions with one other.

Root-associated *Ascomycota* may thrive in stressed environments because their mycelia have a complex structure of fungal cell walls that contain chitin and melanin and are resistant to microbial degradation (2, 7). As a result, *Ascomycota* mycelia are more likely to leave a large proportion of a fungal necromass that serves to store carbon in boreal soil (6, 9). Our results showed that the abundance of mycorrhizal fungi of the phylum *Ascomycota* increased with higher TC contents in the *Sasa*-intact plot (Fig. 6B); however, *Ascomycota* species richness was low. If the abundance of this mycorrhizal fungus is parallel to its greater biomass, one possible explanation for the greater TC in the SI plot is a close relationship between the presence of *S. kurilensis* and the mycorrhizal family *Pezizaceae* (phylum *Ascomycota*). According to our results, *S. kurilensis* appeared to promote the presence of the mycorrhizal family *Pezizaceae* (phylum *Ascomycota*), and the existence of this fungi appeared to increase the TC content in root-free soil. Further studies are needed in order to confirm this theory.

In addition, recent studies have shown that the mycorrhizal helper bacteria of herbaceous plants cooperate with mycorrhizal fungi owing to the presence of trehalose (10, 11). The results of the present study imply that some species of the phylum *Planctomycetes* co-occur with mycorrhizal fungi in *S. kurilensis* rhizomes, according to our analysis of the number of OTUs (Table 3). This result suggests that fungi and bacteria co-occur as a result of a biological interaction. However, since some significant relationships were detected between the phyla present and environmental factors, this co-occurrence may be an artefact due to mutual dependence on the same environmental factors.

## Conclusion

Our results indicate that the presence of *S. kurilensis* in a *B. ermanii* boreal forest affected the compositions of soil fungal and bacterial communities and the corresponding soil properties. More soil water was retained after *S. kurilensis* was removed. Low fungal species richness was found in the *Sasa*-intact and *Sasa*-removed plots. The presence of *S. kurilensis* appeared to promote the colonization of soil mycorrhizal fungi of the family *Pezizaceae* (phylum *Ascomycota*), which may have meditated the amount of soil carbon in the dry soil environment. The present study enriches our understanding of how the presence of understory bamboo affects soil properties and corresponding microbial communities in a boreal forest.

## Figures and Tables

**Table 1:** Alpha diversity estimates of microbial communities in SI (*Sasa*-intact) and SR (*Sasa*-removed) plots for five sampling dates.

	Dates	Number of OTUs^a^		Chao1^b^		Shannon^c^		Simpson^d^		Coverage^e^	
				
		SI plot	SR plot	SI plot	SR plot	SI plot	SR plot	SI plot	SR plot	SI plot	SR plot
Bacteria	23 Jul 2014	847 (64)	852 (15)	999 (52)	1071 (44)	**7.6 (0.1)**	**7.2 (0.1)**	0.99 (0)	0.98 (0)	0.99 (0)	0.99 (0)
	1 Oct 2014	960 (37)	863 (55)	1141 (42)	1021 (49)	7.6 (0.1)	7.5 (0.2)	0.99 (0)	0.99 (0)	0.99 (0)	0.99 (0)
	3 Jun 2015	935 (31)	955 (33)	1146 (37)	1138 (42)	7.8 (0)	7.8 (0.1)	0.99 (0)	0.99 (0)	0.99 (0)	0.99 (0)
	4 Aug 2015	919 (68)	962 (48)	1107 (90)	1155 (49)	7.7 (0.2)	7.7 (0.1)	0.99 (0)	0.99 (0)	0.99 (0)	0.99 (0)
	1 Oct 2015	1043 (43)	998 (22)	1207 (45)	1201 (45)	7.8 (0.1)	7.8 (0.1)	0.99 (0)	0.99 (0)	0.99 (0)	0.99 (0)

Fungi	23 Jul 2014	42 (1)	40 (7)	43 (2)	42 (7)	2.6 (0.4)	2.7 (0.2)	0.72 (0.06)	0.74 (0.02)	1 (0)	1 (0)
	1 Oct 2014	47 (2)	36 (4)	50 (2)	43 (6)	3.2 (0.1)	2.1 (0.7)	0.77 (0.04)	0.55 (0.14)	1 (0)	1 (0)
	3 Jun 2015	43 (5)	46 (5)	44 (6)	48 (6)	2.8 (0.4)	3.7 (0.3)	0.76 (0.07)	0.87 (0.03)	1 (0)	1 (0)
	4 Aug 2015	50 (5)	46 (4)	53 (5)	46 (5)	2.8 (03)	2.9 (0.7)	0.76 (0.06)	0.70 (0.16)	1 (0)	1 (0)
	1 Oct 2015	46 (2)	44 (4)	46 (2)	44 (5)	2.2 (0.1)	3.0 (0.5)	0.62 (0.01)	0.73 (0.13)	1 (0)	1 (0)

Values represent mean (SD) (*n*=3). Values in bold denote a significant difference between the SI (*Sasa*-intact) and SR (*Sasa*-removed) plots (*p*<0.05; one-way ANOVA), otherwise no significant difference.

a Species level, a 97% similarity threshold was used to define operational taxonomic units (OTUs).

b Chao1: Chao1 estimator with species richness.

c Shannon: Shannon Index.

d Simpson: Simpson Index.

e Coverage: Coverage *C* was calculated as *C*=1− (*s*/*n*), where *s* is the number of unique OTUs and *n* is the number of individuals in the sample.

**Table 2:** Percentage (%) of sequences classified for two fungal classes, *Pezizomycetes* and *Tremellomycetes*, in SI (*Sasa*-intact) and SR (*Sasa*-removed) plots for five sampling dates.

Dates		SI plot					SR plot				
	
		23 Jul 2014	1 Oct 2014	3 Jun 2015	4 Aug 2015	1 Oct 2015	23 Jul 2014	1 Oct 2014	3 Jun 2015	4 Aug 2015	1 Oct 2015
Class	*Pezizomycetes*										
Order	*Pezizales*	100	100	100	100	100	100	100	100	100	100
Family	*Pezizaceae*	100	100	100	100	100	100	100	100	100	100

Class	*Tremellomycetes*										
Order	*Filobasidiales*	100	100	100	98	100	99	100	99	98	99
	*Tremellales*	0	0	0	2	0	1	0	1	2	1
Family	*Filobasidiaceae*	100	100	100	98	100	99	100	99	98	99
	*Incertae sedis*	0	0	0	2	0	1	0	1	2	1
Genus	*Cryptococcus*	100	100	100	98	100	99	100	99	98	99
	Unknown spec.	0	0	0	2	0	1	0	1	2	1

**Table 3:** Relationship between soil properties and the number of OTUs of main phyla in SI (*Sasa*-intact) and SR (*Sasa*-removed) plots based on a generalized linear mixed model. The response variable was the number of OTUs of the main phyla. The fixed effect was the concentration of soil properties, and the random effect was the study plot. Only significant results with *p*<0.05 were listed.

Phylum	Soil properties	Intercept	Estimate	SE	*z*-value	*p*
SI plot						
*Firmicutes*	Ammonium	−2.17	0.11	0.03	3.77	<0.001
*Planctomycetes*	Total nitrogen	−1.47	0.34	0.15	−2.23	0.03
	Total carbon	−1.44	0.03	0.01	−2.58	0.01
SR plot						
*Firmicutes*	pH	−0.46	−0.37	0.18	−2.09	0.04
*Proteobacteria*	Total nitrogen	−1.84	0.61	0.27	2.27	0.02
*Basidiomycota*	Ammonium	−0.75	0.01	0	2.08	0.04
